# Mixed-methods pilot feasibility single-arm trial of Beyond Fertility: a brief face-to-face psychosocial intervention to promote patients’ adjustment to the end of unsuccessful fertility treatment

**DOI:** 10.1186/s40814-026-01778-x

**Published:** 2026-02-07

**Authors:** Mariana Sousa-Leite, Raquel Costa, Bárbara Figueiredo, Sofia Gameiro

**Affiliations:** 1 School of Psychology, Cardiff University, Cardiff, UK; 2 EPIUnit ITR, Institute of Public Health of the University of Porto, Porto, Portugal; 3 Lusófona University, HEI‐Lab: Digital Human‐Environment Interaction Labs, Lisbon, Portugal; 4 Psychology Research Centre (CIPsi), School of Psychology, University of Minho, Braga, Portugal

**Keywords:** In vitro fertilisation (IVF), Unsuccessful fertility treatment, Psychosocial end-of-treatment care, Preventive care, Early interventive care

## Abstract

**Background:**

Infertility is a major public health issue with a 17.5% estimated lifetime prevalence. Despite advances in assisted reproductive technology (ART), almost half of those seeking ART end all treatment cycles without a child. Around 93% of patients want to be prepared and supported when facing unsuccessful treatment. Healthcare professionals perceive a high demand for such support, yet evidence-based interventions remain limited. This mixed-methods study evaluated the acceptability and feasibility of implementing and evaluating Beyond Fertility: a brief psychosocial intervention to promote patients’ adjustment to the end of unsuccessful fertility treatment. Results will inform the design of a definitive randomised controlled trial (RCT) of Beyond Fertility.

**Methods:**

This is a prospective, mixed-methods, single-arm pilot study. The Beyond Fertility intervention offers preventive care (one individual/couple session before patients start their last treatment cycle) and early interventive care (one individual/couple and five group sessions if treatment is unsuccessful) to promote patients’ emotional and social adjustment to the end of unsuccessful treatment. Beyond Fertility’s development integrated feedback from patients and healthcare professionals. Adults scheduled to start their last NHS-reimbursed in vitro fertilisation/intracytoplasmic sperm injection (IVF/ICSI) cycle, including the last transfer with own or donated gametes/embryos or preimplantation genetic testing (PGT)—at a large NHS hospital in Portugal were consecutively recruited. Participants completed online questionnaires at baseline (T1, pre-intervention), post-individual/couple sessions (T2), and post-intervention (T3). A post-intervention focus group (T3) gathered feedback on the intervention and the study protocol. Participants also completed online open-ended feasibility questions after each intervention session. Feasibility outcomes focused on demand, acceptability, implementation, practicality, and promise of efficacy. Exploratory analysis of efficacy was change in quality of life (FertiQoL, T1–T3).

**Results:**

Thirty-two participants consented to participate, completed T1, and were allocated to Beyond Fertility (62.1% acceptance rate). Participants reported that preventive care was acceptable, feasible, and beneficial. After a negative cycle outcome, 30.0% (*n* = 9) of participants ended treatment, and most (*n* = 21, 70.0%) continued. Of those who ended treatment, most (*n* = 6, 66.7%) accepted the interventive care, reporting it helped them accept their unmet desire for children and pursue alternative life goals. One-third (*n* = 3, 33.3%) received all sessions. Reasons for non-acceptance or withdrawal from the intervention in the immediate aftermath of treatment were the emotional burden of unsuccessful treatment (33.3%) and the sessions’ group format (22.2%). Facilitators of session uptake were flexible online/in-person scheduling and delivery. Individual quality-of-life trajectories suggested a possible recovery after unsuccessful treatment.

**Conclusions:**

Beyond Fertility was seen as adequate and valued, and it showed promise in improving patients’ quality of life after unsuccessful treatment. Before efficacy testing, feasibility issues require changes to Beyond Fertility’s logic model, including activity design and revisions to its evaluation to target a larger, more representative sample of participants.

**Supplementary Information:**

The online version contains supplementary material available at 10.1186/s40814-026-01778-x.

## Key messages regarding feasibility

What uncertainties existed regarding the feasibility?

- Uncertainties included whether patients accept and engage with preventive support to prepare them for the possibility of an undesired treatment outcome while still undergoing treatment, whether they engage with and value support after ending unsuccessful treatment, and what the optimal timing and delivery format of this support are. Uncertainties about the trial design included whether eligible patients starting their ‘last’ treatment cycle can be identified and retained for follow-up.

What are the key feasibility findings?

- Most patients (~60%) accepted and engaged with preventive care for the end of treatment, having valued and perceived that they benefited from discussing the possibility of treatment not working while undergoing treatment and being supported through its end when unsuccessful. Patients provided positive feedback regarding Beyond Fertility, including its delivery mode and format, and valued such support. However, the acceptability of Beyond Fertility interventive care (as offered) was average-low in the immediate aftermath of unsuccessful treatment due to the emotional impact of experiencing loss (33%) and to some rejection of its group format (22%). Retention in the research was problematic because most patients underwent more cycles when confronted with a negative outcome of their ‘last’ funded cycle.

What are the implications of the feasibility findings for the design of the main study?

- Changes to Beyond Fertility following feedback should take place before progressing to a larger-scale study. Modifications will be made to Beyond Fertility’s logic model, including activity design, to improve acceptability. Recruitment will be expanded to include more fertility clinics, with active engagement of clinic staff to support the integration of Beyond Fertility into routine practice and to encourage patient participation. Conservative a priori sample size calculations based on this pilot study's estimates will be used, and adjustments to the inclusion process after unsuccessful treatment are needed to include more eligible patients. A 6-month follow-up will be included to assess whether long-term changes in quality of life are sustained over time.

## Background

Infertility constitutes a global public health issue [1], with an estimated overall pooled lifetime prevalence of 17.5% [2]. It was considered one of the most prevalent conditions associated with moderate-to-severe disability under age 60 [3]. Those with infertility are increasingly using assisted reproductive technology (ART), particularly in vitro fertilisation/intracytoplasmic sperm injection (IVF/ICSI), to have children [4]. However, nearly half (45.7%) of patients who start treatment end it (i.e. all treatment cycles) without a live birth [5]. This event triggers intense grief associated with moderate to large impairments in well-being and mental health that can protract up to 20 years after treatment ends [6]. Practice guidelines and regulatory bodies stress the need to address the lack of evidence-based support for patients ending unsuccessful treatment [7–9]. Mixed-methods survey research showed nine in 10 patients want to be prepared for this potential outcome and supported in its aftermath [10, 11]. Two self-guided psychosocial interventions to support heterogeneous groups of people with an unmet desire for children improved users’ well-being or mental health [12, 13]. However, to our knowledge, no intervention exists to support patients through the event of unsuccessful treatment, hereafter referred to as end-of-treatment care.

We developed Beyond Fertility, the first face-to-face (online, in-person) psychosocial intervention aiming to promote patients’ adjustment to the end of unsuccessful fertility treatment [11]. Beyond Fertility’s development followed the Medical Research Council (MRC) recommended methodology for developing complex interventions [14, 15]. Supplementary Fig. S1 presents the theoretically informed causal logic of Beyond Fertility. Its design and evaluation process integrated patients’ and healthcare professionals’ feedback via qualitative focus groups [11]. The design was informed by the Three Tasks Model of adjustment to unmet parenthood goals (3TM) [6], as this framework synthesises quantitative and qualitative research on the psychosocial processes underlying a healthy adjustment to the end of unsuccessful fertility treatment [6]. It was also informed by contextual cognitive behavioural therapeutic (CCBT) principles, in particular Acceptance and Commitment Therapy (ACT) and self-compassion [16, 17], as these currently gather the most convincing, high‐quality evidence of leading to effective psychosocial interventions and constitute an adequate framework for targeting the 3TM mechanisms of change [16, 18]. Beyond Fertility encompasses preventive end-of-treatment care to prepare patients for the possibility of unsuccessful treatment, aiming to buffer its negative impact (if it happens). It also encompasses early interventive end-of-treatment care delivered in the immediate aftermath of treatment, aiming to promote patients’ adjustment to this event. The session’s activities target skills to help patients build acceptance towards their unfulfilled wish for children (i.e. recognition, and active involvement with the situation and its negative emotions without attempts to change its frequency or shape), find meaning in the fertility journey (i.e. re-evaluation of the situation and past efforts to deal with it, life values and priorities), develop and commit to (new) valued life goals and foster relational communication and support and a sense of social connectedness. Beyond Fertility is expected to attenuate the negative impact of unsuccessful treatment, being translated into improved well-being and mental health. Quality of life was considered the primary efficacy outcome, as it is often conceptualised under well-being and considered by some as the ultimate measure of healthcare quality [19]. This study reported on a prospective, single-arm pilot trial to explore the acceptability and feasibility of implementing Beyond Fertility in the clinic setting. Given the lack of interventions and patients’ dissatisfaction with current support [6, 7, 10, 20], findings can constitute foundational knowledge for developing innovative support tools.

Beyond Fertility is a brief, highly structured, and goal-oriented intervention designed for easy integration into routine clinical care [21]. Considering its group format and possible online delivery, it is expected that Beyond Fertility can be integrated into clinical workflows and overcome challenges in care delivery related to time, workload, and lack of communication and counselling resources [21]. Face-to-face psychosocial interventions in fertility care have good acceptance and adherence rates but do not target the end of treatment and are delivered exclusively in individual/couple or group formats [22, 23]. Interventions targeting end-of-treatment patients have moderate participation (58.3%) but variable adherence rates (5.6–66.7%), although likely due to challenges with the self-guided format [12, 13]. Previous evaluation of Beyond Fertility’s acceptability and related research showed that patients want end-of-treatment support [10, 11, 20, 24–26], but that engagement can be emotionally challenging [10, 11]. This same research identified implementation challenges. Due to the numerous work stressors, HCPs encounter in their daily routine (e.g. high workload, lack of time) and treatment idiosyncrasies (e.g. long waiting lists, cycles in the private sector, gametes/embryo donation, treatment add-ons), setting an acceptable and effective recruitment method can be challenging [11].

This trial investigated the uncertainties of implementing Beyond Fertility and its evaluation design in a clinical setting, assessing the feasibility of conducting a definitive randomised controlled trial (RCT) of Beyond Fertility. We assessed five dimensions of feasibility that, according to Bowen, Kreuter [27], should be evaluated to identify (1) engagement with Beyond Fertility (i.e. demand), (2) reactions to Beyond Fertility (i.e. acceptability), (3) extent to which Beyond Fertility and its evaluation design were delivered as planned (i.e. implementation), (4) barriers to engagement (i.e. practicality), and (5) promise of efficacy. A focus group after intervention delivery was included to gather an in-depth view of patients’ experiences with Beyond Fertility and the overall study process [28, 29]. Results will inform whether Beyond Fertility should progress to efficacy testing and the design of a definitive RCT.

## Methods

### Design and setting

This was a prospective, small-scale pilot feasibility trial, non-randomised and single-arm, conducted at a large NHS hospital in Portugal. The protocol included three assessments: T1 (baseline): 1 day before the first session (pre-exposure to the intervention), T2: within 1 week after the second session (post-exposure to the individual/couple sessions), and T3: within 1 week after the group sessions (post-exposure to the intervention). Feasibility questions were asked after each session. A 1-h semi-structured focus group followed the T3 assessment. Where applicable, this report follows the CONSORT statement for pilot and feasibility trials [30] and the Guidelines for reporting non-randomised pilot and feasibility studies [31]. Reporting on the qualitative focus group research follows the COREQ framework [32] (see Supplementary Table S1).

### Participants

Eligible participants were adults scheduled to start, within 1 month, their last NHS-reimbursed IVF/ICSI cycle, including the last transfer with own or donated gametes/embryos or preimplantation genetic testing (PGT). Based on the low-threshold approach of Beyond Fertility (i.e. brief, accessible, non-invasive) and controlled study setting, exclusion criteria were having been diagnosed with a mental health problem within the last 2 years, currently receiving therapy, and being unable to read/speak Portuguese.

### Materials

#### Intervention: Beyond Fertility

Supplementary Table S2 describes Beyond Fertility using the Template for Intervention Description and Replication (TIDieR) checklist [33]. Beyond Fertility must be delivered by mental health professionals. Each session consists of structured activities designed to trigger the theorised mechanisms of change (acceptance, meaning-making, the pursuit of new life goals, and perceived social support and relational quality). Preventive care comprises one individual/couple session within 1 month of the last stimulated/thawed cycle. This session aims to facilitate acceptance of one’s unmet desire for children by informing patients and preparing them for the possibility of unsuccessful treatment [10]. Early interventive care comprises one individual/couple session 1 to 2 weeks after the end of unsuccessful treatment, followed by five weekly group sessions. These sessions are directed at facilitating acceptance by triggering self-compassion [17] and cognitive defusion [34, 35], facilitating social support and relational quality by promoting a sense of social connectedness [36, 37] and couples’ communication and support (when participants have a partner) [38], facilitating meaning-making (i.e. finding meaning in the fertility journey) via positive reappraisal [39] and value clarification [34, 35], and facilitating the development and commitment to new valued life goals [34, 35]. The final session is designed to review the therapeutic process, learn skills, and encourage the maintenance of positive change. Beyond Fertility is expected to ameliorate the immediate negative impact of ending unsuccessful treatment and to lead to improvements in quality of life, mental health, and well-being within 6 months of treatment ending, compared with those receiving their usual care at clinics.

### Outcomes

#### Feasibility outcomes

Feasibility outcomes followed Bowen, Kreuter’s [27] framework, and targeted demand, acceptability, implementation, practicality, and promise of efficacy. All materials (including the focus group script) are described in Table 1. Outcomes were considered separately for the intervention (i.e. Beyond Fertility) and its evaluation design. To decide about progression to efficacy evaluation, feasibility outcomes were evaluated and interpreted contextually.

**Table 1:** Feasibility outcomes and assessment for the Beyond Fertility intervention and its evaluation design

Intervention: Beyond Fertility
Demand. Proportion of participants who were eligible to receive the intervention, who accepted to receive it (i.e., intention to use), and who engaged with each therapeutic activity (i.e., perceived demand). Reasons for non-participation and withdrawal over the course of the intervention (related to the intervention)
Acceptability. Responses to a set of three anonymous online open-ended questions about the appropriateness of each therapeutic activity (after receiving it): the most appreciated aspects of the session, the least appreciated ones, and additional comments or suggestions
Implementation. Whether all the planned tasks for each therapeutic activity were delivered as planned (self-reported by the interventionist delivering the intervention). Mental healthcare professionals’ performance while and confidence in implementing the intervention: the degree to which they felt confident implementing each activity (using a Likert-scale from 0: *no confidence at all* to 10: *totally confident*)
Practicality. Duration of the sessions. Proportion of participants who received the intervention as planned (factors that facilitated its delivery and reasons for not going as planned)
Promise of efficacy. Changes across time in participants’ exploratory efficacy outcome (quality of life) were measured at T1, T2, and T3
Study evaluation design
Acceptability. Proportion of participants who completed the informed-consent form and each of the three assessment moments. Reasons for non-participation and withdrawal over the course of the intervention (related to the assessments)
Implementation. Whether the recruitment procedures were followed as planned. Whether assessments were sent to participants on time. Reported issues relating to study procedures or materials
Practicality. Time taken to answer each assessment. Factors affecting the implementation of the evaluation design
Overall study process
All dimensions—Focus groups. A semi‐structured script (Supplementary Material) was developed following existing guidelines [40, 41] to evaluate participants’ experiences with the overall study process (intervention and its evaluation design). Open questions were informed by Bowen, Kreuter’s [27] feasibility framework and covered: demand (experiences in the study), acceptability (satisfaction, perceived appropriateness of the therapeutic activities and materials), implementation (barriers and facilitators to engagement), and practicality (recruitment, time between sessions, format, ease of filling out the questionnaires). Participants were prompted for additional suggestions or comments. A final set of questions based on Mentimeter (interactive audience engagement platform) included the description, in one small sentence, of the participant experience in the study, to rate the extent to which Beyond Fertility helped them adjust to the end of unsuccessful treatment (from 1: *did not help at all* to 7: *was an essential help*), if they would engage with Beyond Fertility again (from 1: *not at all* to 7: *totally*), whether they would recommend Beyond Fertility to a friend (from 1: *I would not recommend it at all* to 7: *I would totally recommend it*), and whether it would be an asset to implement Beyond Fertility in fertility clinics (not at all, yes maybe, yes totally)

#### Questionnaires

Questionnaires were posted on Qualtrics (Qualtrics, Provo, UT, USA). Participants’ background, fertility history (at T1), and changes across time in participants’ mechanisms of change and outcomes (at T1, T2, T3) were assessed. As this was an exploratory feasibility trial, only the exploratory efficacy outcome (Quality of life) will be reported. After each Beyond Fertility session, participants were sent an anonymous questionnaire containing three additional open-ended questions on the intervention’s acceptability (see Table 1).

##### Background

Participants were asked about their age (in years), gender, nationality, place of residence (village or city), education, occupational status, and financial difficulties (‘during the past 12 months, how often… have you had difficulties paying your bills?’ and ‘…have you not had enough money to buy food, clothing, or other things that your family needed?’, from 1: never to 4: very often; a single variable was computed based on the mean of participants’ answers). They were additionally asked about their relationship status, partner’s sex, relationship duration (if applicable), and the number of biological, adopted, or stepchildren.

##### Fertility treatment history

Participants were asked about the age at which they started trying to have a child spontaneously (if applicable) and the age at which they first sought medical help. They were asked how long they had been undergoing treatment, what previous treatment they had undergone, and whether they had children from the treatment (no, yes). They were asked whether they had received specialised psychosocial care (‘throughout your life, have you sought/received psychological support?’), for how long, whether it was due to fertility-related issues, and whether they perceived it as helpful (‘do you consider this support helped you?’ no, yes).

##### Quality of life (exploratory efficacy outcome)

Perceived quality of life was assessed using the core module of FertiQoL [42, 43], which measures quality of life in the context of a fertility problem. This questionnaire covered four life domains: emotional (e.g. ‘do your fertility problems make you angry?’), mind–body (e.g. ‘are you bothered by fatigue because of fertility problems?’), relational (e.g. ‘have fertility problems strengthened your commitment to your partner?’), and social (e.g. ‘do you feel your family can understand what you are going through?’). Scores range from 0 to 100; higher values indicate better quality of life [Cronbach’s α = 0.83 (T1), α = 0.89 (T2), α = 0.82 (T3)].

### Procedure

Women (primary contact in the hospital) were screened consecutively and invited via phone to participate with their partners (if they had one; March–May 2021). Those interested were emailed an information sheet and informed consent form. Participants who provided informed consent, completed the T1 assessment, and were allocated to Beyond Fertility (sessions delivered face-to-face, online or in-person at the hospital, as per participants’ preferences). Participants for whom the stimulated/thawed cycle did not result in pregnancy and ended treatment met the inclusion criteria to receive the early interventive care. The others were offered a referral to specialised support or other support sources. After the second individual/couple and the last group session, participants were sent T2 and T3 assessments, respectively. After each session, participants received a link to anonymously evaluate the session (i.e. acceptability questions about the intervention). Reminders were sent via email and phone. At the end of the study, all participants who initially consented were invited to a focus group. An information sheet and consent form were sent to those willing to participate. The focus group discussion was carried out via the Zoom videoconferencing platform on December 15, 2021. It was moderated by another female psychologist (S.G., PhD) who had no prior relationship with participants, to ensure a comfortable environment. S.G. is a registered psychologist and a researcher with a background in psychosocial reproductive care, as well as extensive experience and training in qualitative interviewing. The focus group was audio-recorded and transcribed verbatim. S.G. kept a reflexive journal throughout the discussion to document field notes, own perceptions, and assumptions. Participants were briefed on the interviewer’s role as a researcher in the study, the study’s aims and procedures, the recording (as per consent), and some ground rules (e.g. confidentiality, welcoming opposite thoughts). At the end, a link to additional feasibility questions (see Table 1) on the Mentimeter platform was sent.

### Data management and analysis

Descriptive statistics were used for the background, the fertility history, the Mentimeter responses, and the exploratory efficacy outcome of quality of life. As is good practice in feasibility evaluation, no inferential statistics were used. Individual total scores and trajectories of quality of life were represented in the data visualisation.

Data from open-ended acceptability questions and the focus group were analysed with thematic analysis [44] using NVivo software version 12 (QSR International). M. S.‐L. and S. G. familiarised themselves with the data and engaged in peer-debriefing discussions about the reflexive notes. M. S.‐L. set the inductive codes (i.e. descriptive meaning labels) for each text segment. The research team (S. G., B. F., R. C., and M. S.‐L.) reviewed the coding and resolved disagreements on interpretation by consensus. Codes were systematically organised into categories. Having Bowen, Kreuter’s [27] feasibility framework as reference, categories were organised into sub-themes and themes (i.e. interpretative descriptions of interrelated ideas). Textual data analysis was presented as a summary accompanied by representative verbatim quotes (translated into English), each referenced by a participant number (P). ‘(…)’ indicated omitted parts of quotes, and ‘[text]’ represented clarifications.

## Results

### Participants

**Fig. 1:**
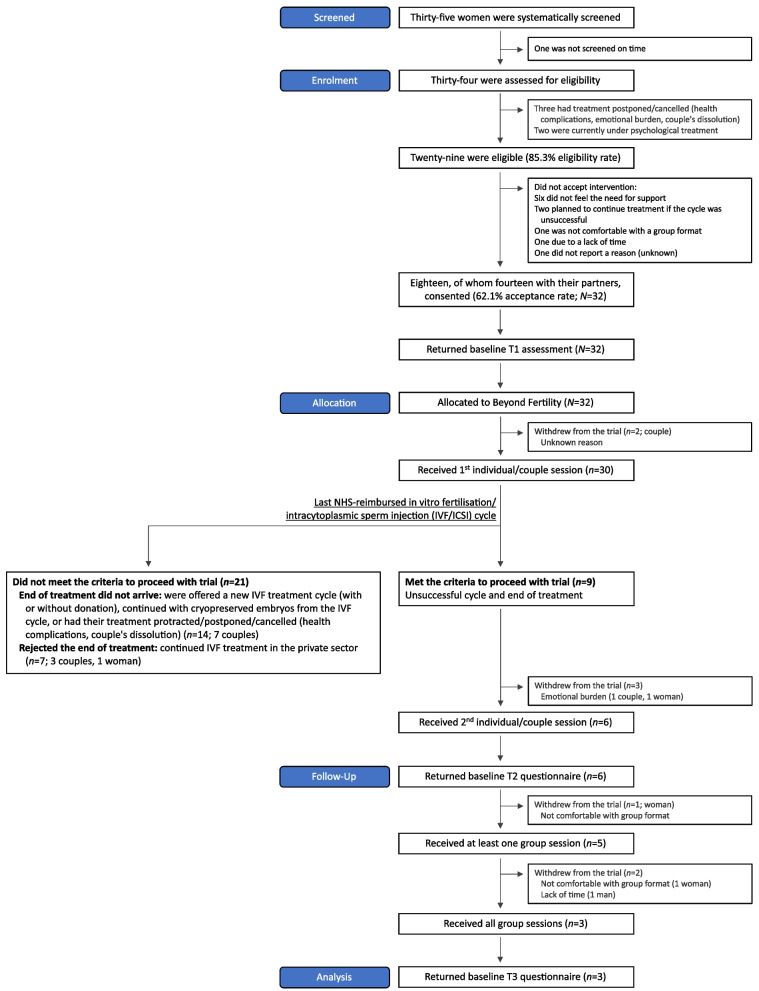
Participant flow diagram

Figure 1 presents the participant flowchart. Thirty-two participants consented (62.1% acceptance rate) and completed the T1 assessment. Table 2 describes participants’ background and fertility history. Participants averaged 38 years of age. Around half were women, two-thirds lived in the city, and half had a university education. Nearly all were employed, and over the past 12 months participants reported experiencing financial difficulties either never or rarely. All were in a heterosexual relationship for an average of 12 years. Over one-third had biological children; none had adopted children, and a minority had stepchildren. Participants started trying to have children on average at 31 years of age and sought medical help on average at 35 years of age. Participants had been undergoing treatment for an average of 2 years. Around half had undergone IVF in the past, averaging 2 cycles, and a minority had children as a result. Around one-third received specialised psychosocial care in the past, on average for 1 year, with a small minority due to fertility-related issues.

**Table 2:** Descriptive statistics of participants’ sociodemographic characteristics, fertility history and psychosocial care received at T1 (baseline) assessment

	Total (*N* = 32)
Sociodemographic characteristics	*n*(%)
Age (years) *M*(*SD*)[interval range]	38.16(3.84)[29.00–45.00]
Women	18(56.25)
Portuguese	29(90.63)
Place of residence	
City	22(68.75)
Village	10(31.25)
University education	16(50.00)
Employed^a^	29(93.55)
Financial difficulties* M*(*SD*)[interval range]	1.06(0.17)[1.00–1.50]
In a heterosexual relationship	32(100.00)
Duration (years) *M*(*SD*)[interval range]	11.58(5.51)[3.00–20.00]
Have children	17(53.13)
Biological	13(40.63)
Adopted	0(0.00)
Stepchildren	6(18.75)
Fertility history	
Age at which started trying to have children spontaneously^b^* M*(*SD*)[interval range]	31.30(5.16)[22.00–38.00]
Age at which sought medical help^b^* M*(*SD*)[interval range]	35.19(4.04)[24.00–42.00]
Duration undergoing treatment^a^ *M*(*SD*)[interval range]	2.24(2.18)[0.00–8.00]
Previous treatments	25(78.13)
Medication	6(18.75)
Surgery	4(12.50)
Artificial insemination	8(25.00)
Number of cycles* M*(*SD*)[interval range]^a^	2.00(0.89)[1.00–3.00]
IVF/ICSI	17(53.13)
Number of cycles* M*(*SD*)[interval range]^a^	1.93(0.96)[1.00–4.00]
Had children from previous treatment	4(16.00)
Received specialised psychosocial care in the past	11(34.38)
Duration (years) *M*(*SD*)[interval range]	1.40(1.45)[0.00–4.00]
Due to fertility-related issues	2(18.18)
Considered it helpful	2(100.00)

*M* = mean; *SD* = standard deviation. Valid percentages were reported (^a^1–2 participants did not report on this variable. ^b^6–7 participants did not report on these variables)

### Feasibility of the Beyond Fertility intervention

#### Demand

Of the 34 women assessed for eligibility, 29 were eligible to participate (85.3% eligibility rate). From these, eighteen women accepted to participate (62.1% acceptance rate). Seventeen, of whom thirteen with their partner, actually participated and received the first session of Beyond Fertility (i.e. preventive care; 58.6% participation rate; *n* = 30). The main reason for non-acceptance was the perception of no need for support. Nine participants (30.0%) ended unsuccessful treatment. Of these, two-thirds (*n* = 6, 66.7%) received at least one session, and 3 (33.3%) received all sessions. The main reasons for withdrawing from Beyond Fertility were the emotional burden of the end of treatment (33.3%) and not feeling comfortable in group (22.2%) in the immediate aftermath of unsuccessful treatment.

#### Acceptability

A total of 74 anonymous responses from 30 participants were collected for the questions evaluating their perceived acceptability of Beyond Fertility. Thematic analysis revealed one main theme and five subthemes, presented in Table 3 with illustrative quotes. The main theme reflected that Beyond Fertility offers appropriate and beneficial support. Themes and sub-themes reflected positive evaluations of Beyond Fertility’s logic model, which was perceived as a valuable tool for end-of-treatment support. Patients reported a high appreciation of the interventionist’s skills, empathy, responsiveness, and expertise in psychosocial fertility care. In general, no disadvantages were perceived from the sessions, but a minority expressed concerns about preventive end-of-treatment care (unnecessarily) triggering negative emotions.

**Table 3:** Themes of acceptability regarding patients’ most and least appreciated aspects of each Beyond Fertility session (*N* = 30)

Theme, description	Illustrative quotes
Positive evaluations of Beyond Fertility’s aims. All patients expressed very positive reactions towards session 1 (i.e., preventive care). Patients appreciated being informed about what most patients experience during the last treatment cycle, what they could expect if the treatment cycle ended unsuccessfully, and how most patients positively adjust to this event (within and beyond parenthood). Only one patient reported that although they appreciated being able to express their emotions, they did not appreciate the session overall. Regarding the sessions after the end of treatment (i.e., early interventive care), patients particularly valued being supported in identifying their emotions, practicing self-compassion, defining and pursuing valued life goals, and practicing mindfulness. Only one patient reported not appreciating the range of tools provided in session four, but no further information was provided	“Without a doubt, the therapist’s thorough clarification (…) indeed, on our drive home, my husband and I found ourselves discussing topics we had never discussed before”, “Reflecting on the positive aspects”, “These sessions help us reflect on things and feelings that may be repressed or hidden, bringing them to the surface and demystifying them as normal (…) As the therapist showed through the dynamics, there is a whole world beyond the pain”, “The deepening of self-compassion”, “The theme of values, understanding that values are different from emotions. Remembering that emotions are truly important to us and how, through them, we can set goals for a fulfilling and happy life beyond motherhood. Ending [the sessions] with mindfulness helps to calm down”, “It was important to discuss our goals after this [therapeutic] process and how we will use what was shared and discussed in the group to move forward”
Positive evaluations of Beyond Fertility’s format. Patients valued the preventive care for offering a space to talk about their feelings and expectations during a moment of uncertainty. They also valued the early interventive care, as it provided support during a time of helplessness, loneliness, and lack of resources. The mix of individual/couple sessions and group support was highly valued. It offered a private one-to-one space for patients to freely share their emotions, fears, and concerns, and a space to share experiences with others going through the same journey, understanding they are not alone and that their experiences are shared	“We really liked this initiative and believe this support is very important”, “Allow us to express how we feel”, “Being able to talk (vent)”, “Being able to openly discuss my situation without any restrictions” [individual format], “Sharing our experiences with others made me feel that I am not alone in these feelings”, “Keep these type of initiatives (…) psychological support is essential, often becoming a safe haven” [early interventive care]
Beyond Fertility helped patients to prepare for the end of treatment and supported them in this transition. Patients reported that the preventive care helped them better understand and feel more prepared to cope with the treatment cycle and possible adverse outcomes. They considered that the early interventive care equipped them with strategies that enabled them to better cope with the end of treatment and contemplate and pursue other paths beyond parenthood	“Being able to clarify doubts that I was hesitant to ask about, but felt comfortable enough to express”, “It strengthened us to face this new chapter of our lives!”, “Sharing [in the group] made us understand that there are several stages of acceptance or even grief”, “It made us realise that we are no different, that the fears and anxieties we feel are common to couples going through the same”, “Finding more strategies to cope with infertility”, “Made us see more clearly the path to follow”
Empathy and expertise of the psychologist. Patients expressed specific positive comments about the psychologist. They considered the psychologist to be very empathic and responsive. Patients felt comfortable and understood sharing their emotions, fears, and concerns, and stressed that it was beneficial to have support from someone outside who was an expert in psychosocial fertility care	“The empathy, attention and knowledge demonstrated by the therapist”, “Empathy, how the therapist made us feel at ease from the start of the session, and their professionalism”, “The understanding and compassion of the therapist”, “In this type of treatment, it is always helpful to speak with someone who can understand us, help us control, and overcome our fears”
No disadvantages to the sessions, but concerns towards preventive end-of-treatment care for a minority. Most patients expressed they could not see any aspects they did not appreciate or appreciated less, repeatedly stressing that psychosocial support throughout treatment is essential. Two patients reported concerns about the preventive care session. Although they appreciated the session, one said it was too focused on the negative side of treatment, whereas they can still be hopeful at this stage. Another said that discussing past and future negative emotions could be challenging and trigger distress in patients. One patient referred to the long duration of the questionnaires	“I don't have anything that I didn't appreciate. Psychological support is essential!”, “I don't feel like there were any less positive aspects”, “Although we understand that the point was to raise awareness that the process may not go well, as we are at the beginning of the IVF process, with all possibilities still open, we feel that too much has been said about the possibility of ending it unsuccessfully”, “Perhaps remembering past feelings and those that will come is always challenging to talk”

A total of 74 anonymous answers

#### Implementation

The interventionist was confident in most sessions except for session 4, which focused on partnership communication and their sexual relationship. Due to the sensitivity of the topic and the limited number of previous group sessions, engaging the group was challenging. Group sessions occurred every 2 weeks instead of weekly to accommodate participants’ availability.

#### Practicality

Individual/couple sessions averaged 1 h and 15 min, and group sessions averaged 2 h. After the end of the cycle, 30% (*n* = 9) of the participants met the inclusion criteria to proceed with the trial (i.e. ended unsuccessful treatment). The other participants (*n* = 21, 70.0%) continued in treatment. This happened because they were either offered a new cycle (with or without donation), continued their current cycle with cryopreserved embryos that resulted from this cycle, or had their treatment postponed or cancelled—mainly due to health complications (*n* = 14, 46.7%), or continued treatment in the private sector (*n* = 7, 23.3%). The small number of participants ending treatment (*n* = 9, 30.0%) made it difficult to schedule the first group sessions consistently, with sessions ranging from 2 weeks to 4 months after treatment. Facilitating implementation factors were the flexibility of online sessions (77.8%) and scheduling outside of working hours.

### Feasibility of the Beyond Fertility evaluation design

#### Acceptability

All 32 participants who consented and started filling out the assessments completed them. Reasons for non-participation or withdrawal were related to the Beyond Fertility intervention, not to the evaluation protocol materials.

#### Implementation

The recruitment procedures were applied as planned. No constraints were reported regarding the implementation procedures or the evaluation protocol materials.

#### Practicality

Participants took an average of 35.04 min (SE = 3.40, range: 12.02–87.38) to complete T1, 21.15 min (SE = 5.42, range: 14.47–37.33) to complete T2, and 48.13 min (SE = 23.90, range: 15.40–94.67) to complete T3.

### Qualitative feasibility evaluation

Of those who initially consented (*N* = 32), seven accepted the focus group invitation (21.9% acceptance rate), and six participated in the focus group discussion (18.8% participation rate). Of these six participants, three received only the preventive care (i.e. the first session of Beyond Fertility): two withdrew after the treatment ended because of the emotional burden, and one had their treatment postponed. The other three received the full intervention. The thematic analysis of the focus group discussion identified one main theme, organised into three themes and eight categories. Table 4 presents the themes and categories with illustrative quotes. The main theme reflected Beyond Fertility’s high demand and acceptability and perceived feasibility challenges. Themes and categories reflected patients' perceptions that Beyond Fertility met their emotional and social needs. The activities, format, and mode of delivery were perceived as adequate and positively evaluated, and the sessions' online delivery and flexible time scheduling facilitated their engagement. Patients reported that a larger group would have been preferable and that the recruitment strategy and materials were doable and adequate, though long.

**Table 4:** Emergent themes from the process evaluation focus group (*n* = 6)

Themes and categories description	Illustrative quotes
Intervention	
Theme: (Demand and Acceptability) Patients considered Beyond Fertility needed and beneficial, meeting a perceived high demand for support	
Category: Demand for holistic and patient-centred support during and, in particular, after the end of treatment. Patients reported that undergoing treatment is emotionally overwhelming, particularly difficult at (and after) the end of treatment. Patients are repeatedly confronted with negative outcomes and feel alone, with a lack of resources to cope with this process. Patients particularly referred to the lack of information about the treatment process and how to receive psychosocial support during and, in particular, after treatment	“I already done 3 treatments, but none of them worked” (Pa3, woman, received first preventive session), “negative thoughts, frustrations, helplessness, the guilt, all of that” (Pa1, woman, received all sessions), “At first [treatment start], everything is new, but then the bump is huge, and the disappointment even bigger” (Pa6, man, received first preventive session), “Nobody talks, nobody. We are in the [waiting] room, aren’t we!? With so 20 couples or more and no one speaks with each other” (Pa1), “We don't have many people to share” (Pa6), “The whole [treatment] process is highly challenging” (Pa4, woman, received first preventive session), “Many times, I didn't know what the next step was, lacking both the information and the space and time (…) This was my biggest challenge throughout the whole process” (Pa4), “We never had attention in terms of mental health, and I think it's essential for the couple” (Pa1)
Category: Very positive reactions were expressed towards Beyond Fertility. Patients considered Beyond Fertility an essential help during the treatment process. Beyond Fertility provided them with a safe place where they felt at ease to share their emotions, fears, and concerns, supported and understood. Patients were grateful for engaging with Beyond Fertility and considered that it should be implemented as routine practice in fertility clinics. Patients also expressed very positive reactions towards the psychologist. They felt they had someone they could turn to, stressing that being an expert in psychosocial fertility care was extremely valuable	“I want to thank [for the sessions]” (Pa2, woman, received all sessions), “It [Beyond Fertility] helped us a lot (…) it was a shame to only have it in the last treatment” (Pa6), “A safe space where we could express our doubts, our anxieties, our fears, our apprehensions (…) that sometimes we might even think that is not normal but is perfectly normal (…) I think this is extremely important” (Pa4), “I think the psychologist was tireless, in the way that she treated us and in the care she provided us, without a doubt that was important during the process” (Pa4), “I also think it was important that we get together and share these issues, talking about these issues with other couples, it also helps” (Pa5, man, received all sessions), “I wish it [Beyond Fertility] can be implemented in the clinics” (Pa1)
Category: Beyond Fertility helped patients to accept and cope with their fertility journey and unmet desire for children. Patients perceived Beyond Fertility eased the psychosocial impact of ending treatment unsuccessfully. Overall, patients considered Beyond Fertility validated and normalised their fears and concerns. They perceived that preventive care provided them with a more comprehensive view of treatment and made them feel supported during this process. Those who received the early interventive care perceived that these latter sessions decreased their feelings of loneliness and helped them better accept their fertility journey and find alternative ways to look at it. Patients reported that Beyond Fertility gave them a new, hopeful outlook towards the future, providing them with a range of coping strategies they perceived they could use beyond the sessions and apply in other challenging life situations	“Reaching the third [cycle], and talking with the therapist, we had more cautious [in terms of expectations]” (Pa6), “In the sense that it [Beyond Fertility] was reassuring throughout the whole process” (Pa4), “Gave us important tools to value other goals beyond fertility and parenthood” (Pa5), “Realising they [concerns, feelings] are normal, right!? I think this gives us a sense of calm and reassurance” (Pa4), “As Pa5 said a moment ago, I think the process was lighter because we could see that other couples were going through the same thing as we and we were not aliens” (Pa1), “For me, these sessions were important to devalue the feeling of guilt a bit (…) the negative thoughts that we were having throughout this process, the difficult memories and how to let go of these negative thoughts, frustrations, helplessness, the guilt, all of that (…) the relationship with the partner, how we interacted” (Pa1), “we learnt a psychological tool, didn’t we? A way of being and thinking that we will end up applying to all domains of our lives” (Pa4)
Theme: (Implementation and practicality) Beyond Fertility’s activities, format, and mode of delivery were perceived as appropriate, although a larger group would have been beneficial	
Category: Beyond Fertility’s activities were appropriate and valuable. Patients considered the aims of each session to be appropriate. Patients perceived that the materials helped them to better understand what was discussed in the sessions. Due to the daily rush, patients reported they did not have the time to work on the additional materials provided at the end of the sessions, but perceived these materials were a resource they had to turn to whenever they felt they needed to or when confronted with future challenging situations (even in other life domains). Patients particularly valued the defusion strategies, self-compassion exercises, and step-by-step guidance on how to set and pursue valued life goals	“The topics addressed were helpful” (Pa1), “Some tools that call my attention were for example, self-compassion, knowing how to value ourselves and knowing how to love ourselves, right!?” (Pa5), “those negative thoughts I had, I spent some moments alone (…) One of the tools I used was acknowledging the thought” (Pa1), “And starting to think about goals and setting small steps at first, then maybe bigger ones, so that we can start working towards those goals and values in a way that makes us feel fulfilled. I think that was important too” (Pa5), “We were always sent the materials, and even in the end, we were sent them all once again, which is great for us. However, sometimes we are caught up at work and with other tasks, and we may not always remember to set aside time to dedicate to this (…) However, in terms of the materials, they were the appropriate ones for sure” (Pa5), “It was the same with us, with the day-to-day rush” (Pa1), “Some of them [therapeutic exercises] I didn't do, but when I did, it made us think about things, not just gloss over everything” (Pa1)
Category: Beyond Fertility’s format and mode of delivery were flexible and adequate. Patients found the individual/couple format ideal, as it helped them build confidence in the therapist, gave them the opportunity to discuss more personal topics, and made them feel more at ease in the group. Regarding this latter format, patients expressed very positive reactions, considering it crucial in the process. Patients valued conducting the sessions online and outside of working hours due to geographic and work constraints. Scheduling the group sessions every two weeks allowed the group to find a time that worked for everyone and gave patients more time to reflect on what was addressed in the session	“Having individual sessions before joining the group let patients feel more at ease, by gradually start talking about these issues individually” (Pa5), “Having sessions every week is too frequent, due to time constraints (…) it gave us time to think and having a weekend in between (…) gave us time for the next session” (Pa1), “From our experience, I think it worked well online. In-person would have been almost impossible (…) there aren't as many constraints [with online format]. It's easier to coordinate, both in terms of schedules and locations where we can be. It's easier for everyone, I think” (Pa1)
Category: Having a larger group would have been valuable for patients. Maintaining the same group throughout the process fostered bonding and trust within the group, but patients perceived they would have benefited more from a larger group	“During the group phase, I found it important that we always kept the same people from the beginning to the end—that was important (…) helped build trust, and people felt confident speaking” (Pa5), “Perhaps having more people could help enrich the group. I understand there were probably constraints, but it's just one aspect that could be improved” (Pa5)
Evaluation protocol	
Theme: (Implementation and practicality) Recruitment strategy was empathic, informative, and appropriate. Although the online assessments were time-consuming, patients considered them comprehensive and easily accessible	
Category: Recruitment strategy was considered appropriate. Patients considered they were empathically invited to participate and adequately informed about the study procedures. They considered they were given the proper time to decide about their participation. No further suggestions were reported	“I think it was well done. The therapist contacted us first and asked if we were interested. We saw what the project was. I think it's the right way” (Pa1), “I also agree with Pa1. We were first contacted by phone, and the entire project was explained to us, and then the information was sent in writing, which allowed us to read it again. I think it was quite appropriate. I really liked it” (Pa4), “I don't see there's another way to do it” (Pa6)
Category: Online assessments were time-consuming, but this was not perceived as a downside. Patients considered that the online format of the assessments facilitated their participation due to accessibility. They also found the questionnaires comprehensive and, although time-consuming, did not consider this to be a factor that would prevent them from participating	“With regard to the surveys, I don't see any problem. If it's online, it's easy to fill out, it's easy to send, they were quite understandable, it was easy to understand and respond” (Pa5), “Sometimes they were a bit long, but that's it, they were manageable, it was only necessary to dedicate some time to it. I suppose it's necessary, so it's part of the process” (Pa5), “I agree with Pa5” (Pa4)

#### Mentimeter results

All the participants of the focus group answered the questions (*n* = 6). Participants considered Beyond Fertility ‘a precious help’ and ‘a rewarding and enriching experience’, helping them adjust to the end of treatment (Mean = 5.8; SD = 0.45; from 1: did not help at all to 7: was an essential help). They would engage with Beyond Fertility again (Mean = 6.8; SD = 0.45; from 1: not at all to 7: totally) and recommend it to a friend (Mean = 6.6; SD = 0.55; from 1: I would not recommend it at all to 7: I would totally recommend it), and all totally agreed that implementing it in clinics would be an asset.

### Descriptives of participants’ individual trajectories on quality of life

#### Quality of life

**Fig. 2:**
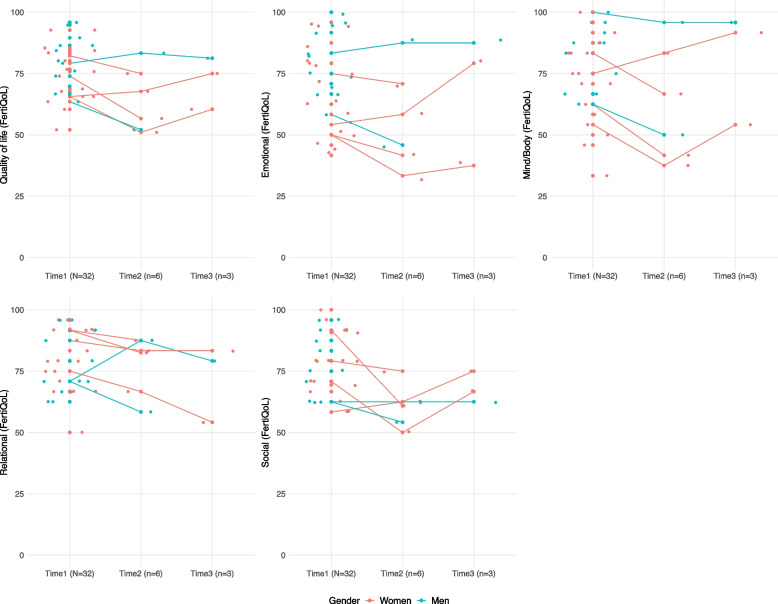
Individual total scores and trajectories on the exploratory efficacy outcome for quality of life and its dimensions. Note. T1 (Baseline: pre-exposure to the beyond fertility intervention), T2 (post-exposure to the individual/couple sessions), and T3 (post-exposure to the group sessions) per gender (women, men). Lines connect the participants who completed both T1 and T2, and those who completed T1, T2, and T3

Individual total scores and trajectories at T1, T2, and T3 for women and men are visualised in Fig. 2. Individual trajectories indicated a decrease in quality of life from T1 to T2 (*n* = 6; more pronounced for women). Trajectories suggested a possible recovery from T2 to T3 (*n* = 2) for women (less pronounced for the social dimension), whereas the man’s trajectory (*n* = 1) appeared to remain stable. This was not observed in the relational dimension for women, where individual trajectories indicated a decrease from T1 to T3. Average scores on total quality of life indicated a decrease from T1 (*N* = 32, mean = 77.91, SE = 2.00, [52.08–95.83]) to T2(*n* = 6, mean = 64.31, SE = 5.39, [51.04–83.33]), and possible recovery at T3 (*n* = 3, mean = 72.22, SE = 6.17, [60.42–81.25]).

## Discussion

Results from this study provide novel evidence that offering preventive end-of-treatment care is feasible and needed, with patients seeing it as adequate and valuable. Combining preventive with early interventive care seems a promising approach to attenuate the negative impact of unsuccessful treatment, but its delivery must be sensitive to patients’ preferences and circumstances. As a model for delivering end-of-treatment care, Beyond Fertility proved more successful in its preventive component. The main barriers to implementation and uptake of the early interventive component were the idiosyncrasies of fertility treatment, complex decision-making about ending treatment, the emotional impact of unsuccessful treatment, and some rejection of the group format. Those who engaged with Beyond Fertility considered it a valuable source of support that helped them accept and manage the impact of unsuccessful treatment and pursue alternative pathways to or beyond parenthood. Adjustments to Beyond Fertility are required.

Providing end-of-treatment preventive care in the form of an individual/couple session was well received and feasible to implement (62.1 acceptance rate and positive evaluations). Only a minority (21%) perceived that end-of-treatment care was not needed. These results confirm previous Beyond Fertility’s acceptability testing [11], other evidence suggesting high demand for end-of-treatment care [6, 7, 10, 20, 26], and patient-reported willingness to discuss negative treatment outcomes in advance [10, 45, 46]. Acceptance of early interventive care in the form of one individual/couple and five group sessions was also high. However, it is important to note that engagement over time was moderate to low, although still higher than observed for self-guided digital support (< 20%) (e.g. 13). This lower engagement seemed to result from patients’ difficulty in managing their negative reactions to their loss in the immediate aftermath of treatment within a therapeutic (33%) and/or group format (22%). Previous research suggested there is an element of readiness to engage with support that is related to one’s ability to let go of their desire for children [13]. This suggests that those in higher need of support [as the strength of a child’s desire is associated with worse adjustment [47] may be less able to engage with it or need more time to feel ready to engage [48, 49]. Clinics could implement patient follow-up processes to enable patients to engage with support when they feel ready and can benefit from it.

Those who engaged with Beyond Fertility found it beneficial. Perceived benefits were consistent with Beyond Fertility’s mechanisms of change, providing further support for the use of the 3TM as a therapeutic end-of-treatment model [6, 13]. For instance, acceptance and meaning-making were perceived as supporting the construction of new views of the world and the redefinition of life priorities [50]. Patients’ feedback on Beyond Fertility’s therapeutic strategies (self-compassion, mindfulness, and pursuit of new valued goals) supports the use of ACT to trigger the 3TM mechanism of change [16, 18, 35]. Considering the specific positive patient feedback on the therapist’s qualities (empathy, expertise), it is possible that the therapeutic relationship may have amplified the impact of the mechanisms of change by increasing patient engagement with and trust in Beyond Fertility [51, 52]. Additional enablers of patient engagement were online and after-hours delivery, which overcame traditional barriers to accessing mental health services [53, 54]. However, this may prove unsustainable across clinics due to limited time, resources, and care integration [21].

Although the patterns observed in individual quality-of-life trajectories should be interpreted as illustrative rather than indicative of treatment effects, they suggest a potential benefit from Beyond Fertility. The observed patterns indicate that preventive care may have a protective effect in ameliorating the negative impact of unsuccessful treatment and that early interventive care may facilitate recovery in the aftermath of treatment, preventing the grief trajectories previously observed in cohort studies [55, 56]. The exception to the trends observed was in relational quality-of-life trajectories, suggesting that Beyond Fertility may be less successful or slower in triggering recovery in the partnership. This is most likely because the therapeutic techniques used, which focused on addressing issues around partnership communication and sexual relationship, were not seen as acceptable in a group format. Overall, the data support the value of proceeding to evaluate Beyond Fertility’s efficacy via a Randomised Controlled Trial after addressing the acceptability and feasibility issues identified.

This pioneering study brings to light potential challenges of implementing and evaluating end-of-treatment care. Specifically, it addresses how to identify patients who are likely to end treatment and how to assess the power to detect individual heterogeneity in patients’ trajectories at this treatment stage. Indeed, only 30% of patients who intended to end treatment if their last funded cycle was unsuccessful actually stopped. The other 70% continued for multiple reasons, including being offered additional cycles not initially planned (e.g. due to poor ovarian response), having surplus cryopreserved embryos, or deciding to continue treatment privately. A better understanding of the heterogeneity in patients’ individual trajectories (possible trajectories, decision-making processes, and outcomes) is needed to support practice and the successful design of end-of-treatment trials.

### Strengths and limitations

This study followed the MRC [14, 15] and feasibility frameworks [27]. Reporting followed the CONSORT statement for pilot and feasibility trials [30]. Guidelines for reporting non-randomised pilot and feasibility studies [31] and the COREQ framework for reporting qualitative research [32]. The in-depth mixed-methods approach strengthened the reliability of the results and the potential efficacy of Beyond Fertility [15, 29]. The systematic recruitment strategy in a large public hospital increased internal validity. Although the study participation rate was low [57], limiting generalisability and conclusions about efficacy and feasibility for a future RCT, the convergence of results with previous research [10, 11] suggests that a larger sample is unlikely to invalidate results. Men’s participation was higher than expected compared with other interventions (e.g. [58]), even though not directly inviting them may have hindered their involvement. It is known that men’s uptake of mental health support is lower when compared with women’s [59]. Considering the lack of end-of-fertility-treatment psychosocial interventions, progression criteria were not defined, as the intention was always to progress to efficacy testing. This limits direct benchmarking of feasibility indicators. However, feasibility outcomes were interpreted contextually, providing explanatory insights to inform optimisation of a future definitive RCT. To standardise patients' experiences, a last treatment cycle was considered as any attempt to initiate a last IVF/ICSI cycle. Patients who had their treatment cancelled or postponed, or who had remaining cryopreserved embryos from this attempt (i.e. did not have a 'complete cycle'), were considered to be facing an unsuccessful cycle, which may have led to an underestimation of the proportion of patients who faced the end of unsuccessful treatment. Participants were representative of white, employed, and heterosexual people, limiting generalisability. Further work is needed with larger, more representative, and cross-cultural samples. In particular, work is needed with patients who undergo treatment as single women or who identify as LGBTQ + community. Considering the low number of participants and data variability, the descriptive statistics of individual trajectories should be carefully considered.

### Implications for future research

Future research should address the limitations mentioned above to further explore the potential of end-of-treatment psychosocial interventions. Findings indicate that investing in end-of-treatment care models that incorporate preventive and early interventive components is valuable and confirms the benefit of targeting specific active components: acceptance, meaning-making, pursuit of new life goals, and perceived social support and relational quality. These findings support the 3TM as an acceptable and potentially effective framework for grounding end-of-treatment support, and the CCBT principles for targeting the 3TM mechanisms of change and guiding Beyond Fertility’s therapeutic activities. Strategies to address the challenges of delivering end-of-treatment care after unsuccessful treatment are needed. Specific to Beyond Fertility, findings suggest revisions to its causal theory to improve feasibility as well as to its evaluation design to improve internal validity and assess outcomes in a larger, more generalisable sample of participants. The following changes will be implemented: for the Beyond Fertility intervention, activities focusing on the partnership (communication, sexual relationship) will be discussed in an individual or couple format (instead of group) to improve the acceptability of the group format. In the evaluation design, a broader recruitment will be considered. First, partnerships with multiple clinics will be established. Second, a priori sample size calculation will be conservative and informed by the estimates from this pilot study. Considering the present eligibility rates of 85.3%, acceptance rates of 62.1%, and intervention completion rates of 9.4%, for a two-arm RCT with quality of life as the primary efficacy outcome, a priori sample size calculations indicated that 340 women should be recruited to achieve a minimum final sample size of 15 participants per group. This would allow detection of a moderate effect-size change in the primary outcome, with a two-sided 5% significance level and a power of 90% [60]. Third, only those patients who are certain of continuing treatment will be excluded from receiving early interventive care. Clinics will have a higher involvement in promoting Beyond Fertility to increase patients’ awareness of the need for psychosocial support and ease access to the intervention. Even though some patients rejected the group format, this will be maintained because it is cost-effective and associated with higher efficacy (than individual/couple format) in fertility care [23]. Other studies document patients’ willingness to receive end-of-treatment care in group format [61].

## Conclusions

This study demonstrates the feasibility of implementing a psychosocial intervention rooted in 3TM and CCBT to promote patients’ adjustment to the end of unsuccessful treatment. Findings indicate a demand for such support and that patients welcome further research on this topic. Results showed that Beyond Fertility is valuable, but additional work is needed to make it fit for delivery after treatment within a preventive and interventive model of care. Considering the positive feedback expressed by patients who received Beyond Fertility, a powered evaluation of its efficacy in promoting patients’ adjustment to the end of treatment seems worthwhile. However, modifications to the intervention logic model, including activity design, and revisions to its evaluation design to target a larger, more representative sample of participants are required.

## Supplementary Information

Supplementary Material 1. Table S1: Consolidated criteria for reporting qualitative studies (COREQ): 32-item checklist

Supplementary Material 2. Table S2: Beyond Fertility description using the TIDieR checklist [33].

Supplementary Material 3. Figure S1: Logic model of the Beyond Fertility psychosocial intervention. Inputs represent the resources used to inform the development of the intervention. Outputs display the planned activities designed to target specific mechanisms of change (psychosocial processes). Outcomes represent the changes that are expected to be seen in real life after the planned activities are reached

## Data Availability

The data underlying this article will be shared upon reasonable request with the corresponding author. The final Beyond Fertility intervention protocol is available on OSF at https://osf.io/ysg5e/.
