# Supplementary material for: Mixed-methods pilot feasibility single-arm trial of Beyond Fertility: a brief face-to-face psychosocial intervention to promote patients’ adjustment to the end of unsuccessful fertility treatment
Source: Pilot Feasibility Stud. 2026 Feb 7;12:36. doi: 10.1186/s40814-026-01778-x (PMC12977741; doi:10.1186/s40814-026-01778-x)
Supplement: Supplementary file 1 — Supplementary Material 1. Table S1: Consolidated criteria for reporting qualitative studies (COREQ): 32-item checklist [file 40814_2026_1778_MOESM1_ESM.docx]

**Supplementary Table 1**

*Consolidated criteria for reporting qualitative studies (COREQ): 32-item checklist*

| **No Item** | **Guide questions/description** | **Reported on Page No.** |
| --- | --- | --- |
| **Domain 1: Research team and reflexivity** | | |
| Personal characteristics | | |
| 1. Interviewer/facilitator | Which author/s conducted the interview or focus group? | 13 |
| 2. Credentials | What were the researcher’s credentials? E.g. *PhD, MD* | 13 |
| 3. Occupation | What was their occupation at the time of the study? | 13 |
| 4. Gender | Was the researcher male or female? | 13 |
| 5. Experience and training | What experience or training did the researcher have? | 13 |
| Relationship with participants | | |
| 6. Relationship established | Was a relationship established prior to study commencement? | 13 |
| 7. Participant knowledge of the interviewer | What did the participants know about the researcher? e.g. *personal goals, reasons for doing the research* | 13 |
| 8. Interviewer characteristics | What characteristics were reported about the interviewer/facilitator? e.g. *Bias, assumptions, reasons and interests in the research topic* | 13 |
| **Domain 2: study design** | | |
| Theoretical framework | | |
| 9. Methodological orientation and  Theory | What methodological orientation was stated to underpin the study? e.g. *grounded theory, discourse analysis, ethnography, phenomenology, content analysis* | 13-14 |
| Participant selection |  |  |
| 10. Sampling | How were participants selected? e.g. *purposive, convenience, consecutive, snowball* | 12 |
| 11. Method of approach | How were participants approached? e.g. *face-to-face, telephone, mail, email* | 12 |
| 12. Sample size | How many participants were in the study? | 14, Figure 1 |
| 13. Non-participation | How many people refused to participate or dropped out? Reasons? | 16-17, Figure 1 |
| Setting | | |
| 14. Setting of data collection | Where was the data collected? e.g. *home, clinic, workplace* | 13 |
| 15. Presence of non-participants | Was anyone else present besides the participants and researchers? | N/A |
| 16. Description of sample | What are the important characteristics of the sample? e.g. *demographic data, date* | 9-10, 14, Table 2 |
| Data collection |  |  |
| 17. Interview guide | Were questions, prompts, guides provided by the authors? Was it pilot tested? | Table 1 |
| 18. Repeat interviews | Were repeat interviews carried out? If yes, how many? | N/A |
| 19. Audio/visual recording | Did the research use audio or visual recording to collect the data? | 13 |
| 20. Field notes | Were field notes made during and/or after the interview or focus group? | 13 |
| 21. Duration | What was the duration of the interviews or focus group? | 9 |
| 22. Data saturation | Was data saturation discussed? | N/A |
| 23. Transcripts returned | Were transcripts returned to participants for comment and/or correction? | N/A |
| **Domain 3: analysis and findings** | | |
| Data analysis | | |
| 24. Number of data coders | How many data coders coded the data? | 13-14 |
| 25. Description of the coding tree | Did authors provide a description of the coding tree? | Table 4 |
| 26. Derivation of themes | Were themes identified in advance or derived from the data? | 13-14 |
| 27. Software | What software, if applicable, was used to manage the data? | 13 |
| 28. Participant checking | Did participants provide feedback on the findings? | N/A |
| Reporting | | |
| 29. Quotations presented | Were participant quotations presented to illustrate the themes / findings? Was each  quotation identified? e.g. *participant number* | Table 4 |
| 30. Data and findings consistent | Was there consistency between the data presented and the findings? | 16-17, Table 4 |
| 31. Clarity of major themes | Were major themes clearly presented in the findings? | 16-17, Table 4 |
| 32. Clarity of minor themes | Is there a description of diverse cases or discussion of minor themes? | Table 4 |
