# Supplementary material for: Mixed-methods pilot feasibility single-arm trial of Beyond Fertility: a brief face-to-face psychosocial intervention to promote patients’ adjustment to the end of unsuccessful fertility treatment
Source: Pilot Feasibility Stud. 2026 Feb 7;12:36. doi: 10.1186/s40814-026-01778-x (PMC12977741; doi:10.1186/s40814-026-01778-x)
Supplement: Supplementary file 2 — Supplementary Material 2. Table S2: Beyond Fertility description using the TIDieR checklist [33]. [file 40814_2026_1778_MOESM2_ESM.docx]

# **Supplementary Table 2**

# *Beyond Fertility description using the TIDieR checklist (33)*

| **BRIEF NAME:** Beyond Fertility: a brief psychosocial intervention to promote patients’ adjustment to the end of unsuccessful fertility treatment. |
| --- |
| **WHY:** Beyond Fertility was developed in response to a current unaddressed need in fertility care, recognised by several international fertility guidelines and regulatory bodies (7-9). It was designed by registered psychologists and researchers, following the Medical Research Council (MRC) framework for developing and evaluating complex interventions (14, 15). Its mechanisms of change (i.e., acceptance, meaning-making, pursuit of new life goals, and perceived social support and relational quality) were informed by the Three Tasks Model of adjustment to unmet parenthood goals (3TM) (6), which hypothesises that targeting these mechanisms promotes patients’ psychosocial adjustment to the end of unsuccessful fertility treatment, translated into better mental health and well-being. The 3TM was evaluated in heterogeneous samples of people with unmet parenthood goals, including those who ended unsuccessful fertility treatment, and showed good acceptability and promising efficacy results in improving users’ well-being (13). Beyond Fertility used Contextual Cognitive Behavioural Therapy (CCBT) principles, in particular from Acceptance and Commitment Therapy (ACT) and self-compassion (16,17), as these gather high‐quality evidence of effective psychosocial interventions and are adequate to target the mechanisms of change proposed by 3TM (16, 18). Based on these principles, the 3TM mechanisms of change were translated into several therapeutic activities implemented in seven therapeutic sessions. |
| **WHAT: Materials:** The interventionist is provided with a comprehensive manual describing each therapeutic session: specific goals, a step-by-step explanation of each therapeutic activity, and the required materials. **Procedures:** At the end of each session, patients are provided with a copy of the materials used during the session and additional materials (with other therapeutic tasks) to practice on their own. At the end of the intervention, patients are provided with a compound of all the materials. |
| **WHO PROVIDED:** The interventionist is a mental healthcare professional (accredited psychologist, psychiatrist, or counsellor trained in psychology). |
| **HOW:** Seven face-to-face therapeutic sessions (in-person or online, depending on patients’ preferences): two individual/couple and five group sessions. |
| **WHERE:** In-person sessions in the fertility outpatient clinic and online sessions via the Zoom videoconferencing platform. |
| **WHEN and HOW MUCH:** The first individual/couple session occurs within one month of the patient’s scheduled date to start their last in vitro fertilisation/intracytoplasmic sperm injection (IVF/ICSI) treatment cycle (i.e., preventive care). The following sessions start within one to two weeks after the end of unsuccessful treatment (i.e., early interventive care): the second in an individual/couple format, plus five in group format occurring weekly. Each individual/couple session has a planned duration of 1h/1:15h, and each group session has a planned duration of 2h. There is no recommended time to engage with the additional therapeutic tasks that patients are given after the session, which they can practice on their own and engage with at repeated times. |
| **TAILORING:** Considering the face-to-face format of Beyond Fertility, there is some level of tailoring from the mental healthcare professional according to the reactions and queries from the individuals/groups during the sessions. |
| **MODIFICATIONS:** Group sessions were scheduled every two weeks (instead of every week as initially proposed) to accommodate participants’ availability and preferences. Group sessions started two weeks to four months after the end of unsuccessful fertility treatment. |
| **HOW WELL: Planned:** Intervention adherence and fidelity outcomes were assessed according to Bowen, Kreuter’s (27) feasibility framework (i.e., demand, acceptability, implementation, and practicality). **Actual:** N/A. |
| *Note*. TIDieR=Template for Intervention Description and Replication. Early interventive care is only directed to those patients who face the end of unsuccessful treatment. |
