# Supplementary material for: Mixed-methods pilot feasibility single-arm trial of Beyond Fertility: a brief face-to-face psychosocial intervention to promote patients’ adjustment to the end of unsuccessful fertility treatment
Source: Pilot Feasibility Stud. 2026 Feb 7;12:36. doi: 10.1186/s40814-026-01778-x (PMC12977741; doi:10.1186/s40814-026-01778-x)
Supplement: Supplementary file 3 — Supplementary Material 3. Figure S1: Logic model of the Beyond Fertility psychosocial intervention. Inputs represent the resources used to inform the development of the intervention. Outputs display the planned activities designed to target specific mechanisms of change (psychosocial processes). Outcomes represent the changes that are expected to be seen in real life after the planned activities are reached [file 40814_2026_1778_MOESM3_ESM.docx]

# **Supplementary Figure 1**

# Logic *model of the Beyond Fertility psychosocial intervention. Inputs represent the resources used to inform the development of the intervention. Outputs display the planned activities designed to target specific mechanisms of change (psychosocial processes). Outcomes represent the changes that are expected to be seen in real life after the planned activities are reached*
